# Supplementary material for: Prevalence and Clinical Profiling of Dysglycemia and HIV Infection in Persons With Pulmonary Tuberculosis in Brazil
Source: Front Med (Lausanne). 2022 Jan 21;8:804173. doi: 10.3389/fmed.2021.804173 (PMC8814308; doi:10.3389/fmed.2021.804173)
Supplement: Supplementary file 1 [file Data_Sheet_1.pdf]

Supplementary Material

Supplementary Table 1. Characteristics of TB cases by glycemic status in RePORT- Brazil cohort

Characteristics	Dysglycemia (n=714)	Normoglycemia (n=558)	p-value
**Male sex – no. (%)**	491 (68.8)	277 (61.8)	**0.018**
**Age – median (IQR)**	39 (29-52)	31 (23-42)	**<0.001**
**HIV infection – no. (%)**	153 (21.5)	91 (20.7)	0.798
**Race/Ethnicity – no. (%)**			**0.007**
White	135 (18.9)	107 (23.9)	
Black	174 (24.4)	128 (28.6)	
Asian	7 (1.0)	0 (0.0)	
Pardo	388 (54.4)	206 (46.0)	
Indigenous	9 (1.3)	7 (1.6)	
**BMI- (kg/m^2) – median (IQR)**	20.5 (18.4-23.1)	19.8 (17.9-22.0)	**<0.001**
**Smoking – no. (%)**	350 (54.6)	224 (50.0)	0.140
**Alcohol consumption – no. (%)**	605 (84.7)	364 (81.2)	0.141
**Illicit drug use – no. (%)**	247 (34.6)	142 (31.7)	0.331
**Positive smear – no. (%)**	542 (76.4)	324 (73.1)	0.232
**Previous diagnosis of diabetes – no. (%)**	128 (17.9)	8 (1.79)	**<0.001**
**Abnormal chest X-ray – no. (%)**	686 (96.1)	430 (96.0)	0.480
**Drug-susceptibility testing (DST) – no. (%)**			
Rifampicin-Isoniazid resistance	16 (2.5)	10 (2.6)	1.000
Rifampicin resistance	18 (2.82)	13 (3.37)	0.756
Isoniazid resistance	52 (8.14)	25 (6.48)	0.392
Sensitive	628 (86.5)	400 (87.6)	1.000
**Symptoms of TB– no. (%)**			
Hemoptysis	151 (24.7)	91 (25.2)	0.924
Cough	613 (95.5)	363 (92.8)	0.096
Fever	509 (79.3)	298 (76.2)	0.280
Weight Loss	597 (93.0)	344 (88.4)	**0.016**
Fatigue	529 (82.4)	311 (79.5)	0.289
Night sweats	452 (70.5)	266 (64.1)	0.476
Chest pain	415 (64.7)	250 (64.1)	0.888

Table note: TB cases were divided in two groups based on the glycemic status in normoglycemia or dysglycemia, which included both diabetes and prediabetes. Data represent no. (%), except for age and BMI, which is presented as median and interquartile range (IQR). Continuous variables were compared using the Mann-Whitney *U* test and categorical variables were using the Fisher's exact test (2x2) or Pearson's chi-square test.

Definition of alcohol consumption: Past or current any consumption of alcohol. *Definition of passive smoking:* Living with someone who smokes. *Definition of illicit drug use:* Past or current illicit drug use (marijuana, cocaine, heroin or crack). *Definition of Pardo ethnicity*: mixture of European, black and Amerindian

Abbreviations: TB: tuberculosis, BMI: Body Mass Index

Supplementary Table 2. Characteristics of TB cases by DM status in RePORT-Brazil cohort

Characteristics	Diabetes (n=275)	Prediabetes (n=439)	Normoglycemia (n=448)	p-value
**Sex male – no. (%)**	182 (66.2)	309 (70.4)	277 (61.8)	**0.027**
**Age – median (IQR)**	46 (36-55)	36 (26-47)	31 (23-42)	**<0.001**
**HIV infection – no. (%)**	56 (20.5)	97 (22.2)	91 (20.7)	0.821
**Race/Ethnicity – no. (%)**				**<0.001**
White	43 (15.7)	92 (21.0)	107 (23.9)	
Black	58 (21.2)	116 (26.4)	128 (28.6)	
Asian	2 (0.73)	5 (1.14)	0 (0.0)	
Pardo	167 (60.9)	221 (50.3)	206 (46.0)	
Indigenous	4 (1.46)	5 (1.14)	7 (1.56)	
**BMI (kg/m^2)-median (IQR)**	21.6 (19.1-24.4)	19.9 (18.4-21.8)	19.8 (17.9-22.0)	**<0.001**
**Smoking – no. (%)**	158 (57.6)	232 (52.8)	224 (50.0)	0.150
**Alcohol consumption – no. (%)**	242 (88.0)	363 (82.7)	364 (81.2)	0.053
**Illicit drug use – no. (%)**	77 (28.0)	170 (38.8)	142 (31.7)	**0.007**
**Positive Smear – no. (%)**	220 (80.3)	332 (74.0)	324 (73.1)	0.077
**TB clinical form– no. (%)**				0.591
Pulmonary	248 (90.2)	388 (88.2)	399 (88.7)	
Pulmonary + Extrapulmonary	27 (9.8)	52 (11.8)	51 (11.3)	
**Previous diagnosis of diabetes – no. (%)**	122 (44.4)	6 (1.4)	8 (1.8)	**<0.001**
**Abnormal chest X-ray– no. (%)**	269 (97.8)	417 (95.0)	430 (96.0)	0.168
**Drug-susceptibility testing (DST) – no. (%)**				
Rifampicin resistance	10 (4.0)	8 (2.1)	13 (3.4)	0.331
Isoniazid resistance	21 (8.4)	31 (8.0)	25 (6.5)	0.608
Rifampicin-Isoniazid resistance	8 (3.2)	8 (2.1)	10 (2.6)	0.666
Sensitive	236 (84.4)	392 (87.8)	400 (87.5)	1.000
**Symptoms of TB– no. (%)**				
Hemoptysis	64 (27.2)	87 (23.1)	91 (25.2)	0.515
Cough	235 (94.0)	378 (96.4)	363 (92.8)	0.083
Fever	196 (78.4)	313 (79.8)	298 (76.2)	0.466
Weight Loss	235 (94.0)	362 (92.3)	344 (88.4)	**0.033**
Fatigue	214 (85.6)	315 (80.4)	311 (79.5)	0.131
Night sweats	174 (69.9)	278 (70.9)	266 (68.2)	0.708
Chest paint	165 (66.3)	250 (63.8)	250 (64.1)	0.796

Table note: Data represent no. (%), except for age and BMI, which is presented as median and interquartile range (IQR). Continuous variables were compared using the Kruskal-Wallis test and categorical variables were using the Pearson's chi-square test. Bold values represent statistically significant.

Definition of alcohol consumption: Past or current any consumption of alcohol. *Definition of smoking:* Past or current cigarette smoker. *Definition of passive smoking:* Living with someone who smokes. *Definition of illicit drug use:* Past or current illicit drug use (marijuana, cocaine, heroin or crack)

Definition of persistence of symptoms: Patients who in the initial evaluation interview (baseline) reported indicated symptom and in the evaluation of visit 2 (month 2) still reported having such symptom.

Definition of Pardo ethnicity: mixture of European, black and Amerindian

Abbreviations: TB: tuberculosis, BMI: Body Mass Index

Supplementary Table 3. Characteristics of TB cases by DM status in SINAN cohort

Characteristics	Diabetes (n=25765)	Normoglycemia (n=253378)	p-value
**Male sex – no. (%)**	16172 (62.8)	171638 (67.7)	**<0.001**
**Age – median (IQR)**	55.0 (46.0-64.0)	40.0 (29.0-54.0)	**<0.001**
**HIV infection – no. (%)**	1051 (4.08)	31073 (12.3)	**<0.001**
**ART use – no. (%)**	484 (11.2)	14175 (27.8)	**<0.001**
**Race/Ethnicity – no. (%)**			**<0.001**
White	8243 (33.8)	77442 (32.4)	
Black/*Pardo*	15750 (64.6)	156749 (65.5)	
Indigenous	181 (0.74)	2997 (1.25)	
Asian	210 (0.86)	1953 (0.82)	
**Abnormal chest X-ray – no. (%)**	20113 (79.3)	190895 (76.7)	**<0.001**
**Alcohol consumption – no. (%)**	3820 (14.9)	45548 (18.0)	**<0.001**
**Illicit drug use – no. (%)**	1116 (4.35)	26696 (10.6)	**<0.001**
**Smoking – no. (%)**	4526 (17.7)	47877 (19.0)	**<0.001**
**Positive smear – no. (%)**	14649 (56.9)	128692 (50.8)	**<0.001**
**TB clinical form– no. (%)**			**<0.001**
**Pulmonary**	23443 (91.0)	217256 (85.7)	
Extrapulmonary	1322 (5.1)	21203 (8.4)	
**Pulmonary + Extrapulmonary**	1001 (3.9)	14919 (5.9)	
**Positive culture – no. (%)**	4527 (17.6)	42879 (16.9)	**0.003**
**Drug-susceptibility testing (DST) – no. (%)**			0.116
Rifampicin resistance	40 (1.8)	428 (2.1)	
Isoniazid resistance	117 (5.4)	1048 (5.1)	
Rifampicin-Isoniazid resistance	113 (5.2)	857 (4.2)	
Sensitive	1906 (87.6)	18211 (88.6)	

Table note: Data represent no. (%), except for age, which is presented as median and interquartile range (IQR). Continuous variables were compared using the Mann-Whitney *U* test and categorical variables were using the Fisher's exact test (2x2) or Pearson's chi-square test. Bold values represent statistically significant

Definition of Pardo ethnicity: mixture of European, black and Amerindian

Abbreviations: TB: tuberculosis, ART: antiretroviral therapy

1 Figure Legends

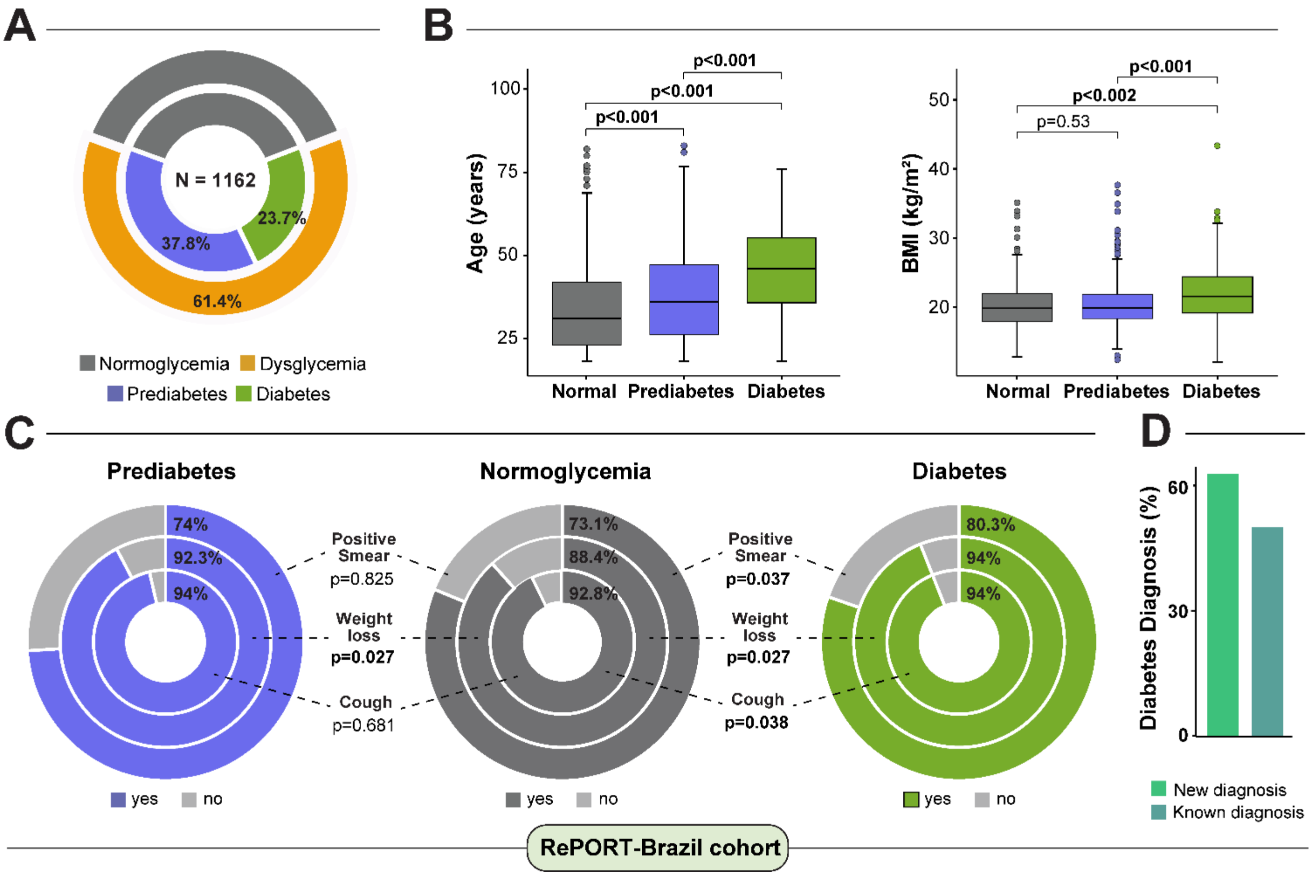

Supplementary Figure 1. Characteristics of patients with pulmonary TB according to dysglycemia status in RePORT-Brazil cohort. **(A)** Among all the individuals with active pulmonary TB (n=1162), 61.4% had dysglycemia: 37.8% pre-DM and 23.7% DM. **(B)** Comparison of Age and BMI between groups were performed using Mann-Whitney *U* test. **(C)** Characteristics of the pulmonary TB cases stratified according to the presence of diabetes or prediabetes were compared with those from patients with normoglycemia using the Fisher's exact test (additional comparisons are displayed in **Supplementary Table 2**). **(D)** Frequency of new cases of DM diagnosis. The statistical analyzes were carried out only with the available data, omitting the cases with missing information. Abbreviations: TB: tuberculosis, BMI: Body Mass Index.

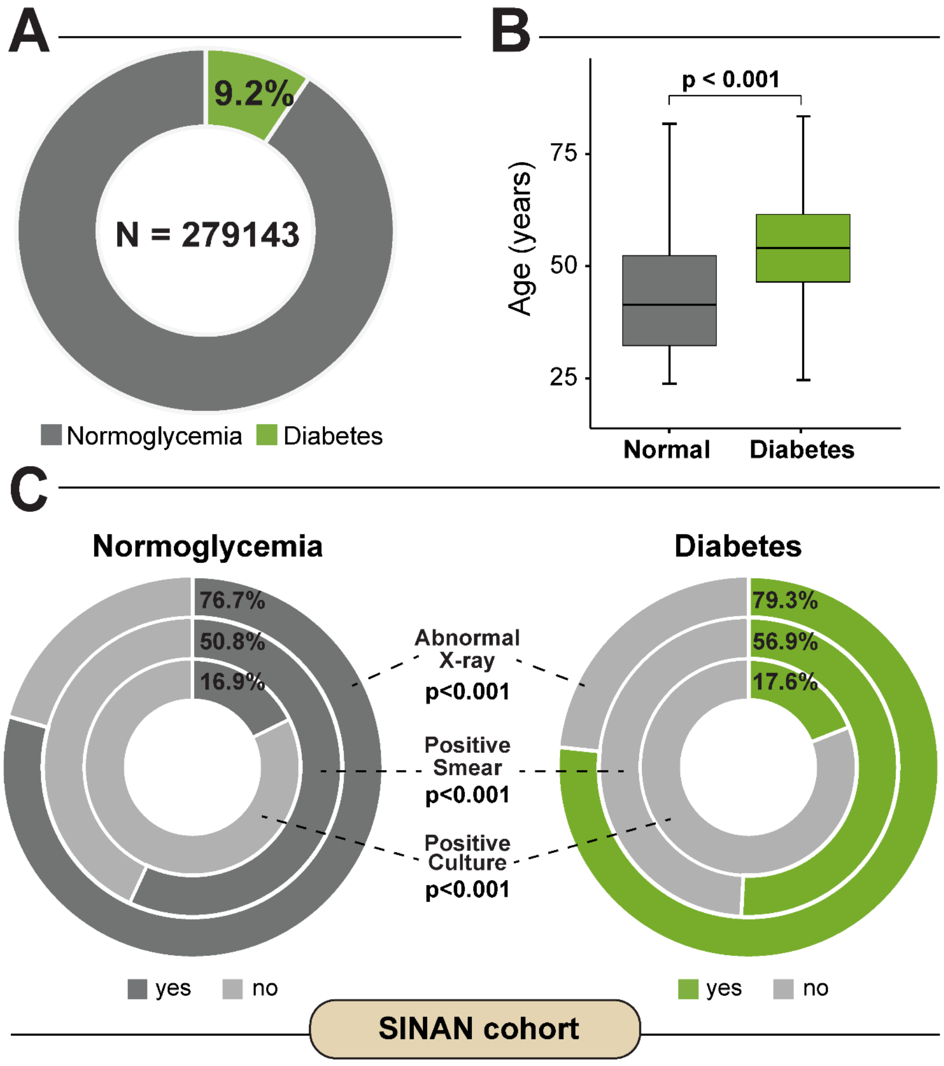

Supplementary Figure 2. Characteristics of patients with pulmonary TB according to DM status in SINAN-TB cohort. **(A)** Among all the individuals with active pulmonary TB (n=287581) between 2015 and 2019 in Brazil, 9.2% had DM. **(B)** Comparison age between groups were performed using Mann-Whitney *U* test. **(C)** Characteristics of the pulmonary TB cases stratified according to the presence of diabetes were compared with those from patients with normoglycemia using the Fisher's exact test (additional comparisons are displayed in **Supplementary Table 3**). The statistical analyzes were carried out only with the available data, omitting the cases with missing information.
